# *In vitro* assessment of Brazilian red propolis against mycobacteria: antibacterial potency, synergy, inhibition of biofilm formation, and intramacrophage effects

**DOI:** 10.3389/fphar.2025.1630134

**Published:** 2025-08-20

**Authors:** S. B. Martins, S. C. Teixeira, G. de Souza, Bader Y. Alhatlani, Emad M. Abdallah, S. R. Ambrosio, T. S. Silva, J. K. Bastos, M. H. Tanimoto, B. F. Barbosa, E. A. V. Ferro, C. H. G. Martins

**Affiliations:** ^1^ Laboratory of Antimicrobial Testing, Institute of Biomedical Sciences, Federal University of Uberlândia – UFU, Uberlândia, Minas Gerais, Brazil; ^2^ Laboratory of Immunophysiology of Reproduction, Institute of Biomedical Sciences, Federal University of Uberlândia – UFU, Uberlândia, Minas Gerais, Brazil; ^3^ Unit of Scientific Research, Applied College, Qassim University, Buraydah, Saudi Arabia; ^4^ Department of Biology, College of Science, Qassim University, Buraydah, Saudi Arabia; ^5^ Nucleus of Research in Technological and Exact Sciences, University of Franca - UNIFRAN, São Paulo, Brazil; ^6^ School of Pharmaceutical Sciences of Ribeirão Preto, University of São Paulo - USP, São Paulo, Brazil

**Keywords:** anti-tuberculous, Brazilian red propolis, antibacterial activity, antibiofilm, health

## Abstract

**Background:**

Tuberculosis persists as a major global health threat and remains the leading cause of death from infectious disease. Efforts to control the disease are increasingly hampered by the emergence of drug-resistant *Mycobacterium tuberculosis* strains. At the same time, non-tuberculous mycobacteria are an expanding clinical concern, with few effective therapies available. Brazilian red propolis (BRP) has shown broad-spectrum antibacterial activity, yet its efficacy against mycobacteria is poorly characterized.

**Methods:**

This study evaluated the *in vitro* antimycobacterial potential of a crude hydroalcoholic extract of BRP (CHEBRP). Minimum inhibitory concentrations were determined against drug-susceptible and rifampicin-resistant *M. tuberculosis* strains (*M. tuberculosis* H37Rv–ATCC 27294, clinical isolate, and rifampicin-resistant clinical isolate; *M. kansasii* ATCC 12478 and clinical isolate; *M. avium* ATCC 25291 and clinical isolate). Fractional inhibitory concentration indices were calculated to assess interactions with isoniazid and rifampicin. Biofilm inhibition was measured, and cytotoxicity was assessed in RAW 264.7 macrophages. Intracellular activity was quantified using infected macrophage cultures.

**Results:**

CHEBRP exhibited potent activity against most *M. tuberculosis* strains tested, including rifampicin-resistant strains. Its combination with isoniazid or rifampicin yielded an indifferent interaction, supporting the feasibility of co-administration. CHEBRP significantly inhibited biofilm formation, showed minimal cytotoxicity toward macrophages, and achieved substantial clearance of intracellular bacilli.

**Conclusion:**

These *in vitro* findings highlight CHEBRP as a promising candidate for adjunctive antimycobacterial therapy. Further studies should investigate its *in vivo* efficacy, pharmacokinetics, and activity against a broader range of mycobacterial species.

## Introduction

Tuberculosis (TB), caused by *Mycobacterium tuberculosis*, remains one of the most fatal infectious diseases globally. According to the latest 2024 WHO Global Tuberculosis Report, TB has re-emerged as the world’s leading infectious cause of death and surpassing even COVID-19. This concerning resurgence emphasizes the ongoing public health challenge posed by TB worldwide. Moreover, it illustrates the significant gap that still exists in reaching the End TB Strategy goals, which include an 80% decline in new TB cases, a 90% reduction in TB-related deaths, and the removal of catastrophic costs for families affected by the disease by the year 2030 (Goletti et al., 2025).

Historically, TB is one of the oldest known infectious diseases, with evidence tracing back to ancient civilizations. Skeletal remains from ancient Egypt, dated to 4000 BCE, exhibit spinal deformities consistent with TB (Zink et al., 2001). Similar pathological signs have been identified in pre-Columbian mummies from Peru (Salo et al., 1994), highlighting its global presence long before the bacterium was scientifically identified. The causative agent, *M. tuberculosis*, was discovered by Robert Koch in 1882, marking a turning point in TB diagnosis and research (Gradmann, 2001).

In 1993, the World Health Organization (WHO) declared tuberculosis (TB) a global public health emergency. In 2014, the World Health Assembly launched “The End TB Strategy,” aiming to reduce TB incidence by 95% by 2035 (World Health Organization, 2015). Despite these efforts, TB-related deaths increased globally and in Brazil in 2022 and the WHO data show TB ranks 13th among all causes of death (World Health Organization, 2022).

Currently, there are more than 190 recognized species of *Mycobacterium*, the only genus in the *Mycobacteriaceae* family, which mostly comprises saprophytic microorganisms found in water and soil. However, some strains are of public health interest because they cause tuberculosis (*M. tuberculosis* Complex) and others, classified as non-tuberculous mycobacteria (NTM) or atypical, are associated with pulmonary, cutaneous and disseminated infections, especially in immunocompromised individuals (Fedrizzi et al., 2017; Armstrong et al., 2023).

Mycobacteria have a peculiar cell wall composed of arabinogalactan and long-chain branched fatty acids called mycolic acids, which confer the property of alcohol-acid resistance observed by Ziehl-Neelsen staining (Holzheimer et al., 2021). Moreover, this structure is related to the survival of bacteria inside macrophages (Huang et al., 2020), with mechanisms of resistance to antibiotics and the ability to aggregate bacteria and form biofilms, which significantly contribute to pathogenicity and increased virulence (Chakraborty et al., 2021).

Indeed, it has been more than 140 years since the German bacteriologist Robert Koch identified *M. tuberculosis* as the etiological agent of tuberculosis (Sakula, 1982), and the disease, one of the oldest and deadliest in humanity, still stands out as a serious challenge for health systems (Aslam et al., 2021).

In Brazil, TB treatment for adults and adolescents involves a 6-month regimen of isoniazid, rifampicin, ethambutol, and pyrazinamide, aiming to rapidly eliminate bacilli and prevent resistance (BRAZIL. Ministry of Health, 2019). However, inadequate or incomplete treatment has led to the emergence of drug-resistant strains, posing a significant global threat (Khoshnood et al., 2021). Resistance has even been observed for newer drugs like bedaquiline and delamanid, approved by the FDA (Mallick et al., 2022).

Similarly, infections caused by NTM species such as *Mycobacterium avium* and *Mycobacterium kansasii*, considered as opportunistic and emerging pathogens in various parts of the world, are difficult to treat and may not respond to medications. Moreover, their ability to form biofilms and their persistence in water systems, pipes, instruments and hospital equipment constitute important sources of contamination (Falkinham, 2021).

Thus, faced with so many challenges and in the search for treatment alternatives, contemporary science has sought solutions in nature and concentrated efforts on the study of bioactive principles derived from natural products. In this logic, propolis has gained notoriety in recent times in various parts of the world, and this can be evidenced by the growing number of publications on its chemical composition and biological properties (Hossain R. et al., 2022; Salatino et al., 2021).

Propolis is a substance produced by bees from resins, gums and balsams collected from plants, mixed with salivary secretions, wax and pollen. The extraction process with hydroalcoholic solvents allows the obtaining of a crude extract whose characteristics, such as aroma, color and flavor, vary according to the botanical origin and concentration of soluble elements (Sforcin and Bankova, 2011).

More than 300 compounds from different types of propolis have already been isolated, especially flavonoids, terpenes and terpenoids (Kasote et al., 2022), and many of them have proven biological activities. Thus, there are many publications demonstrating the antibacterial (Vadillo-Rodríguez et al., 2021), antioxidant (Nichitoi et al., 2021), antiparasitic (Sousa et al., 2023), antifungal (Sallemi et al., 2022), antiviral (Magnavacca et al., 2022), antitumor (Pereira et al., 2021) and immunomodulatory (Conte et al., 2021) potential of propolis, which can even act synergistically and intensify the action of other drugs commonly used in medical practice (Regueira et al., 2017).

According to the chemical composition, physical aspects and regions of the country where they are found, Brazilian propolis was divided into twelve classes (Park et al., 2002) until, in 2007, independent works (Trusheva et al., 2006; Silva et al., 2008) chemically characterized a new type, Brazilian red propolis (BRP), while another group identified its main botanical origin as being *Dalbe*rgia *ecastaphyllum*, which occurs especially in the mangroves of Northeastern Brazil (Daugsch et al., 2008).

Recently, phytochemical and chromatographic analyses confirmed that *Symphonia globulifera* L. f. (Clusiaceae) is also a secondary plant source, contributing with a variety of chemical constituents to the composition of BRP, mainly polyprenylated benzophenones (Ccana-Ccapatinta et al., 2020).

The antibacterial potential of BRP, attributed to the flavonoids that constitute it (mainly isoflavonoids and prenylated benzophenones), has already been confirmed against various types of bacteria (Boeing et al., 2021; De Souza Silva et al., 2021). However, the search in databases for articles related to the antibacterial potential of the product against the genus *Mycobacterium* revealed a lack of studies in this area.

Given the above, the importance of discovering new drugs with antimycobacterial capacity is clear and BRP, being composed of substances with great antibacterial potential already demonstrated in studies involving other genera of bacteria, has stood out in this sense (Maiolini et al., 2020). However, a research of the literature revealed no prior *in vitro* or *in vivo* studies evaluating the antimycobacterial activity of Brazilian red propolis.

Accordingly, the current study aimed to evaluate the antimycobacterial potential of the crude hydroalcoholic extract of Brazilian red propolis (CHEBRP), focusing on its activity against planktonic *Mycobacterium* cells and its ability to inhibit biofilm formation *in vitro*. Additionally, the potential synergistic effects of CHEBRP in combination with standard antimycobacterial drugs were examined. Given the capacity of mycobacteria to evade immune responses and persist within macrophages, the study also investigated CHEBRP’s efficacy against intracellular bacilli. Overall, the research seeks to support the development of novel therapeutic agents derived from natural products, offering alternative mechanisms of action and potentially reduced adverse effects compared to conventional synthetic treatments.

## Materials and methods

### Extraction of brazilian red propolis

**FIGURE 1:**
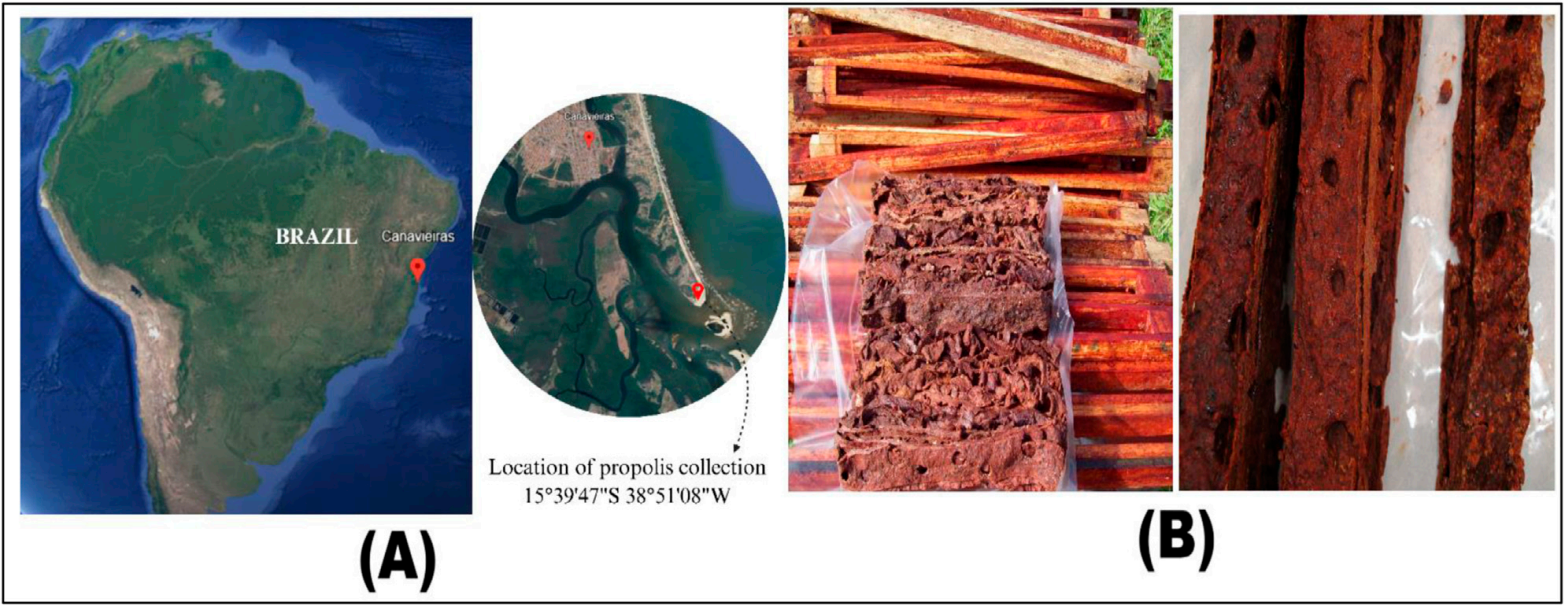
Origin and Collection Site of the Brazilian Red Propolis. **(A) **The collection site. **(B)** The collected samples.

Red propolis raw material was obtained from the Cooperative of Beekeepers of Canavieiras (COAPER) in Bahia, Brazil. The collected Brazilian red propolis and its collection site are shown in (Figure 1). This study was registered with the National System for Management of Genetic Heritage and Associated Traditional Knowledge (SisGen) under registration number AF234D8. One kilogram of red propolis was placed in Erlenmeyer flasks and mixed with a hydroalcoholic solution (ethanol/water 7:3). After soaking for 2 hours, the propolis was ground using a mixer-type processor. The solution volume was then adjusted to a 1:5 (g/mL) ratio. The flasks were shaken in an incubator shaker (Innova 4300TM) at 120 rpm and 37°C for 24 h. The mixture was subsequently filtered through filter paper. This extraction process was repeated three times. The combined extracts were concentrated under vacuum using a rotary evaporator with a temperature set below 40°C. The resulting extract was lyophilized and analyzed using high-performance liquid chromatography with a diode array detector (HPLC-DAD) to determine its chromatographic profile.

### HPLC-DAD analysis

The crude extract was analyzed using HPLC-DAD, following the developed method from previous studies (Aldana-Mejía et al., 2021; Ccana-Ccapatinta et al., 2020). The extract was prepared in HPLC-grade methanol, filtered through PTFE filters (0.45 µm) and stored in vials. Analysis was performed on a Waters^®^ 2695 HPLC system with binary pumps, an automatic injector and a diode array detector, managed by Empower three software. The stationary phase used a Supelco Ascentis Express C18 column (2.7 μm × 150 mm × 4.6 mm) coupled with an analytical Synergi Polar-RP pre-column (4.0 × 3.0 mm, 4 μm). The mobile phase consisted of water acidified with formic acid (0.1%) and HPLC-grade acetonitrile, with a flow rate of 1 mL/min and a 10-µL injection volume. The gradient elution started with 20% acetonitrile, increased to 50% over 40 min, reached 100% by 90 min, was held until 95 min and returned to 20% by 100 min. Detection spanned 210–600 nm, focusing on 280 nm for chromatogram A. The identification of the major compounds was performed according to Aldana-Mejía et al. (2021) and Wolfender (2009). The goal was to present the chemical profile of the major compounds, providing valuable insights that may guide future approaches.

### Commercial drugs

Isoniazid (INH) and rifampicin (RIF) were acquired from Sigma-Aldrich (St. Louis, MO, USA). Stock solutions were prepared according to the manufacturer’s instructions, initially at a concentration of 10,000 μg/mL, aliquoted and kept frozen until the time of use.

### Mycobacteria strains

*M. tuberculosis* H37Rv (ATCC 27294), *Mycobacterium avium* (ATCC 25291) and *Mycobacterium kansasii* ATCC 12478) were purchased from the American Type Culture Collection (ATCC, Manassas, VA, USA).

Clinically isolated strains of *M. tuberculosis*, *M. avium* and *M. kansasii* were kindly supplied by the Adolfo Lutz Institute (São Paulo–SP) and are part of the culture collection of the Laboratory of Antimicrobial Testing (LEA), maintained under cryopreservation at −80°C.

*M. tuberculosis* strain with resistance to RIF (TB RR) was detected through the Rapid Molecular Test for Tuberculosis (TRM-TB) using the GeneXpert System (Cepheid), originating from a clinical isolate at the Clinical Hospital of the Federal University of Uberlandia. It was grown in Löwenstein-Jensen medium and generously provided by the Microbiology Service of the Clinical Analysis Laboratory Unit of the same institution.

All strains were subcultured in Ogawa-Kudoh culture medium (Laborclin, Pinhais, PR, Brazil) and incubated at 37°C for 15 days until they reached the exponential growth necessary for the assays.

### Raw 264.7 cell culture and maintenance

Murine macrophage-like Raw 264.7 cells (ATCC TIB-71) were cultured in 25-cm^2^ flasks using RPMI 1640 medium (Cultilab, Campinas, SP, Brazil) supplemented with penicillin at 100 U/mL, streptomycin at 100 µg (both Sigma Chemical Co., St Louis, MO, USA) and 10% fetal bovine serum (FBS) (Cultilab, Campinas, Brazil). Cell culture was incubated in a humidified atmosphere of 5% CO_2_ at 37°C. Cell culture maintenance was carried out by serial passaging every 2 days, using a cell scraper and replacing the RPMI-FBS medium (Aldana-Mejía et al., 2021).

### Determination of Minimal Inhibitory Concentration (MIC)

The MIC is the lowest concentration of an antimicrobial, measured in μg/mL, that completely inhibits the growth of bacteria under specific *in vitro* conditions within a predetermined time frame (CLSI, 2023). This research assessed the antibacterial efficacy of CHEBRP against *Mycobacterium* species using the broth microdilution method with Middlebrook 7H9 medium (Difco, Detroid, USA). Serial dilutions of CHEBRP, INH and RIF were conducted in sterile 96-well polystyrene microplates. To prevent desiccation during incubation, the outer wells were filled with sterile distilled water, and controls for bacterial growth and broth sterility were included. The experiment was conducted in triplicate.

In summary, CHEBRP was dissolved in 5% dimethyl sulfoxide (DMSO) to obtain the following concentrations: 31.25 μg/mL, 62.5, 125, 250, 500, 1,000 and 2,000 μg/mL. The inoculum was prepared by transferring a range of colonies grown in Ogawa-Kudoh (Laborclin, Pinhais, PR, Brazil) medium into a tube containing glass beads with 500 μL of sterile water. A 200-μL portion was then transferred to a tube containing 2 mL of Middlebrook 7H9 medium supplemented with oleic acid, bovine albumin, sodium chloride, dextrose and catalase (OADC), and incubated at 37°C for 7 days, being subsequently compared with a McFarland scale of 1. Following this, the inoculum was adjusted to reach a cell density of 6 × 10^6^ CFU/mL.

Standard antibiotics INH and RIF were employed at concentrations ranging from 0.015 to 1.0 μg/mL, except for the TB RR strain, for which the final concentration of the antibiotic rifampicin was 200 μg/mL. The plates were then incubated at 37°C for 7 days. After the incubation period, 30 μL of a 0.01% resazurin solution was added to each well to assess bacterial growth. The plates were reincubated at 37°C for an additional 24 h before reading. Color change was indicative of viability following the REMA protocol (Palomino et al., 2007).

### Checkerboard synergy assay

The synergistic effects of combinations of CHEBRP with INH and RIF were evaluated by fractional inhibitory concentration index (FICI) for each of the tested mycobacteria. Checkerboard assays, based on the method described by Bhusal et al. (2005) with modifications, were conducted in triplicate on 96-well plates. The strains tested included 3 *M. tuberculosis* strains (ATCC, IC, and TB RR), as well as *M. kansasii* ATCC and a clinical isolate due to their higher MIC results. Initially, serial dilutions of CHEBRP were prepared in 100 μL of 7H9 OADC broth in columns 1 of the plates (wells from rows A to G), starting at four times the MIC value for each mycobacterial strain. Similarly, serial dilutions of the antibiotics INH and RIF were prepared in wells from rows H (columns 3–11), starting at four times the MIC. Combined MIC was performed in wells from columns 2 to 11, rows A to G, containing 50 μL of 7H9 broth. For CHEBRP, 50 μL of a concentration eight times the MIC was added to the first row of each column, followed by vertical serial dilutions. For antibiotics, dilutions were performed in ten test tubes, with the first tube containing a concentration four times the MIC of each strain. Then, 50 μL from tube one was added to wells in column 2 of each plate up to row G. The process continued with subsequent tubes, with 50 μL transferred to columns 3 and so forth until tube 10, from which the same volume was added to column 11. Subsequently, 100 μL of standardized inocula (prepared as in the MIC assay) were added to all wells, and plates were incubated at 37°C for 7 days. Afterwards, 30 μL of 0.1% resazurin solution was added, and the plates were reincubated at 37°C for an additional 24 h before reading. FICI were calculated using the formula FICI = (MIC A+ B/MIC A) + (MIC B+ A/MIC B), where MIC A+ B represents the Minimum Inhibitory Concentration of drug A in combination with drug B, and MIC B+ A represents the MIC of drug B in combination with drug A. MIC A and MIC B represent the MIC of drugs A and B when tested individually. Interpretation of results followed these criteria: FICI ≤0.5 indicated synergy; FICI >0.5 and <1, additive effect; FICI ≥1 and <4, indifference; and ICIF ≥4, antagonism (Lewis, 2002).

### Inhibition of biofilm formation

The assay aimed to determine whether CHEBRP could inhibit monospecies biofilm formation. Strains with MIC ≤125 μg/mL from *Mycobacterium* species were used, cultured in Ogawa Kudoh medium at 37°C for 15 days. The procedure closely mirrored MIC determination for planktonic cells, employing microdilutions of CHEBRP in 96-well plates containing Middlebrook 7H9 broth. INH served as the standard control, with similar wells used as positive controls. The assays were conducted in triplicate, assessing both biomass and metabolic activity of the biofilm across different plates.

After incubation of the plates, wells were washed to remove planktonic cells, preserving the biofilm. The biofilm was fixed with methanol, stained with crystal violet, and then solubilized with 95% ethanol. Spectrophotometric reading at 570 nm was conducted to interpret the results. Biofilm formation was considered when the absorbance was equal to or greater than 1.0 (Carter et al., 2003). The percentage of inhibition was calculated using the formula: Inhibition (%) = 100 - (average absorbance of treated wells x 100)/average absorbance of control wells (untreated). Through the analysis of the results, it was possible to obtain the Minimum Inhibitory Concentration of Biofilm (MICB_50_), defined as the lowest concentration of EBPVB that showed 50% or more inhibition of biofilm formation compared to the positive control (Wei, 2006).

To assess the metabolic activity of mycobacterial biofilms after exposure to CHEBRP, the MTT (bromide of (3-(4,5-dimethylthiazol-2-yl)-2,5-diphenyl bromide tetrazolium)) dye technique was used as a cellular viability indicator. MTT is reduced by enzymes in live cells, forming insoluble crystals that can be measured spectrophotometrically. After incubating the plates, the supernatant was carefully removed from the wells without damaging the biofilms. Subsequently, an MTT solution (5 mg/mL in PBS buffer) was added, and the plate was incubated again. After the reaction, the liquid was aspirated and replaced with DMSO to solubilize the formed crystals (Montoro et al., 2005). The absorbance was measured at 490 nm. The reduction in mycobacterial viability within the biofilm was calculated by comparing the absorbance of treated and untreated wells using the same formula as described in the biomass reduction assay above.

### Cytotoxicity test

The cytotoxic effect of CHEBRP on murine macrophages, Raw 264.7, was evaluated based on the protocol described by Islam et al. (Islam et al., 2021) with modifications. Initially, cells were cultured in 96-well plates (1 × 10^4^ cells/well) containing 200 µL of RPMI medium supplemented with 10% fetal bovine serum and incubated overnight at 37°C in 5% CO_2_. Upon reaching 80% confluence, wells were washed with PBS and treated with eight concentrations of CHEBRP ranging from 512 to 4 μg/mL (1:2 dilutions), with the assay performed in triplicate.

Wells containing only cells with culture medium and cells with solvent (DMSO) were used as controls, while INH, at concentrations ranging from 25 to 0.19 μg/mL, was employed as the standard antibiotic. After 72 h, 100 µL of MTT reagent at 0.5 mg/mL was added to each well and incubated in a CO_2_ incubator at 37°C for 4 h. After this time, the medium was aspirated, and the formazan crystals were solubilized with 100 µL of DMSO. Finally, absorbance was measured at 570 nm (Titertek Multiskan Plus, Flow Laboratories, McLean, VA, USA), and the optical densities (OD) obtained were converted into percentage cell viability by comparison with the control group according to the formula: % Viability = (OD Treated Group x 100)/Mean OD Control Group (Montoro et al., 2005).

### Intracellular anti-mycobacterial activity

Raw 264.7 macrophages were exposed to a multiplicity of infection (MOI) of 10:1 of *M. tuberculosis* ATCC and TB RR. Inoculums of 1 × 10^5^ CFU/well were prepared following the MIC test. Raw 264.7 cells were cultured in RPMI medium supplemented with 10% fetal bovine serum and adjusted to 1 × 10^4^ cells/100 μL/well in 96-well plates, incubated for 24 h at 37°C in a 5% CO_2_ atmosphere. The cultures were then infected and incubated for 2 hours to allow bacilli internalization, followed by washing with phosphate-buffered saline (PBS) to remove non-phagocytized mycobacteria. Subsequently, cells were treated with CHEBRP and INH at concentrations equivalent to the MIC and ½ MIC of each strain and incubated for 72 h at 37°C in CO_2_. Controls included wells with untreated infected cells and infected cells treated only with DMSO. After 3 days, the wells were washed again with PBS, the macrophages were lysed with 0.2% Triton X-100, and bacterial viability was determined by luminescence assay (Islam et al., 2021).

### Luminescent microbial cell viability assay

The cell lysates were transferred to a 96-well opaque-walled plate. Bacterial viability assay was performed using the BacTiter-Glo™ kit (Promega), following the manufacturer’s instructions. Six determinations were carried out for each control, CHEBRP and INH at different concentrations. Control wells containing only medium (without cells) were used to obtain the luminescence background, which was read on a GloMax Explorer luminometer after 1 s of exposure.

### Statistical analysis

All data were expressed as means ± standard deviations (SD). Statistical analysis was performed using Graph Pad Prism 8.0 (Graph Pad Software, Inc., CA, USA). One-way ANOVA was used for analysis of variance and multiple comparisons between the means of experiments, complemented by Tukey’s test. Non-parametric data were analyzed using the Kruskal–Wallis test and Dunn’s multiple comparison test. Differences were considered significant when P < 0.05.

## Results

### Extraction and HPLC analysis of brazilian red propolis

The extraction of Brazilian red propolis yielded a dry, intensely reddish extract with an approximate yield of 48% relative to the original raw material. High-performance liquid chromatography with diode-array detection (HPLC-DAD) revealed a complex chromatographic profile consistent with the characteristic chemical markers of red propolis, including flavonoids, isoflavonoids, pterocarpans, and benzophenones (Figure 6).

The chromatogram exhibited well-defined and reproducible peaks, confirming the efficiency of the extraction method in preserving bioactive compounds. The triplicate extraction procedure enhanced recovery, as demonstrated by the consistent chromatographic profiles of individual batches and the final pooled extract. Lyophilization resulted in a stable and concentrated material suitable for biological assays and pharmaceutical applications.

The HPLC-DAD chromatographic analysis of the Brazilian red propolis extract revealed the presence of several major bioactive compounds. Within the isoflavonoid class, the compounds liquiritigenin, vestitol, neovestitol, and 7-O-neovestitol were identified. Additionally, the pterocarpan derivative medicarpin was detected, along with two benzophenones: guttiferone E and oblongifolin A. All the compounds presented well-defined and reproducible peaks, confirming the effectiveness of the extraction method in preserving chemical integrity.

### Evaluation of *in vitro* antimycobacterial activity and synergistic effects

The antimycobacterial activity of CHEBRP was assessed both individually and in combination with the reference antibiotics isoniazid (INH) and rifampicin (RIF) by determining the Minimal Inhibitory Concentration (MIC) and employing the Fractional Inhibitory Concentration (checkerboard assay), respectively. The MIC values of CHEBRP ranged from 31.25 μg/mL to 250 μg/mL. The best results were observed against *M. tuberculosis* strains: H37Rv (ATCC 27294) and a clinical isolate, both with MIC of 62.5 μg/mL. Notably, the rifampicin-resistant strain (TB RR) showed the lowest MIC among the tested mycobacteria (31.25 μg/mL). Regarding *Mycobacterium avium* (ATCC 25291) and a clinical isolate, both exhibited identical MICs of 250 μg/mL, as did *Mycobacterium kansasii* (ATCC 12478) and a clinical isolate, with MIC values of 125 μg/mL. The minimum inhibitory concentrations of CHEBRP and antibiotics are shown in Table 1.

**TABLE 1:** MIC (μg/mL) results of CHEBRP, INH and RIF against mycobacteria.

Strains	CHEBRP	INH	RIF
*M. avium* (ATCC 25291)	250	>1.0	0.06
*M. avium* (Clinical Isolate)	250	0.25	0.03
*M. kansasii* (ATCC 12478)	125	0.5	0.125
*M. kansasii* (Clinical Isolate)	125	0.5	0.125
*M. tuberculosis* (ATCC 27294)	62.5	0.25	0.06
*M. tuberculosis* (Clinical Isolate)	62.5	0.5	0.06
*M. tuberculosis* RR (Clinical Isolate)	31.25	0.06	>200

The results obtained for the Fractional Inhibitory Concentration Index (FICI) after evaluating the interaction between CHEBRP and the antibiotic INH, as demonstrated in Table 2, revealed values ranging from 2.0 to 4.0. These data did not show synergistic or antagonistic effects, meaning the association was indifferent to the tested mycobacterial strains.

**TABLE 2:** Determination of the synergistic activity of CHEBRP in combination with INH against *Mycobacterium* strains.

Strains	MIC alone		MIC combined					
	(µg/mL)		(µg/mL)		FIC		FICI	Outcome
	CHEBRP	INH	CHEBRP	INH	CHEBRP	INH		
*M. tuberculosis* (ATCC 27294)	62.5	0.25	62.5	0.25	1.0	1.0	2.0	indifferent
*M. tuberculosis* (Clinical Isolate)	62.5	0.5	62.5	0.5	1.0	1.0	2.0	indifferent
*M. tuberculosis RR* (Clinical Isolate)	31.25	0.06	62.5	0.03	2.0	0.5	2.5	indifferent
*M. kansasii* (ATCC 12478)	125	0.5	250	1.0	2.0	2.0	4.0	indifferent
*M. kansasii* (Clinical Isolate)	125	0.5	125	1.0	1.0	2.0	3.0	indifferent

Similarly, for the antibiotic RIF, the FICI results indicated that the interaction with CHEBRP resulted in indifferent effects, with values ranging between 1.5 and 4.0, as shown in Table 3. In the case of the *M. tuberculosis* RR strain, known to be resistant to RIF, no synergy test was conducted with this antibiotic.

**TABLE 3:** Determination of the synergistic activity of the CHEBRP in combination with RIF against *Mycobacterium* strains.

Strains	MIC alone		MIC combined					
	(µg/mL)		(µg/mL)		FIC		FICI	Outcome
	CHEBRP	RIF	CHEBRP	RIF	CHEBRP	RIF		
*M. tuberculosis* (ATCC 27294)	62.5	0.06	62.5	0.03	1.0	0.5	1.5	indifferent
*M. tuberculosis* (Clinical Isolate)	62.5	0.06	62.5	0.06	1.0	2.0	2.0	indifferent
*M. kansasii* (ATCC 12478)	125	0.12	250	0.24	2.0	2.0	4.0	indifferent
*M. kansasii* (Clinical Isolate)	125	0.12	250	0.06	2.0	0.5	2.5	indifferent

### Inhibition of biofilm formation

The antibiofilm activity of CHEBRP and INH, utilized as the control antibiotic, was evaluated by assessing the reduction in biomass. This evaluation involved determining the MICB_50_, which represents the lowest concentration of antimycobacterial agents that exhibited 50% or more inhibition of biofilm formation. Additionally, a cell viability test was conducted using MTT. Overall, for all analyzed mycobacteria, significant reductions in the biomass of monospecies biofilms were observed compared to untreated controls. For *M. tuberculosis* ATCC and clinical isolate, the MICB_50_ of CHEBRP, with 62.5 μg/mL, coincided with the MIC results obtained for planktonic cells and decreased the biomass amount by 60.6% and 59.6%, respectively. The MICB_50_ values for *M. tuberculosis* RR, *Mycobacterium kansasii* ATCC and the clinical isolate of *M. kansasii* were 125, 500 and 250 μg/mL, respectively. These values, along with the other concentrations of CHEBRP and their corresponding percentages of inhibition of mycobacterial biofilm formation assessed through the crystal violet biomass determination test, are presented in Table 4. The MICB_50_ values were highlighted with stripes for easier identification.

**TABLE 4:** CHEBRP concentrations and their inhibitory effectiveness (%) on mycobacterial biofilm formation.

*M. tuberculosis* (ATCC 27294)		*M. tuberculosis* Clinical Isolate		*M. tuberculosis* RR Clinical Isolate		*M. kansasii* (ATCC 12478)		*M. kansasii* Clinical Isolate	
(μg/mL)	(%)	(μg/mL)	(%)	(μg/mL)	(%)	(μg/mL)	(%)	(μg/mL)	(%)
31.25	49.50	31.25	34.76	31.25	29.28	31.25	36.97	31.25	45.14
*62.5	60.58	*62.5	59.63	62.5	45.22	62.5	38.62	62.5	47.78
125	61,66	125	61.61	*125	66.93	125	48,88	125	49.88
250	61.90	250	68.39	250	71.60	250	49.05	*250	76.98
500	66.0	500	69.50	500	72.95	*500	72.84	500	78.67
1	69.25	1	71.38	1	75.55	1	76.62	1	83.03
2	70.72	2	73.10	2	78.02	2	79.00	2	84.62

Isoniazid, a crucial drug in the treatment of tuberculosis and other mycobacterial infections, demonstrated a high capacity for inhibition *in vitro* of biofilm formation and reduction of bacterial metabolism across all strains, even at the lowest concentrations employed in the assays. For *M. tuberculosis* ATCC and TB RR, the MICB_50_ values were 0.06 μg/mL, with a reduction in biomass of 59.7% and 63.6%, respectively. The MICB_50_ values for the clinical isolate strain of *M. tuberculosis*, *Mycobacterium kansasii* ATCC and the clinical isolate of *M. kansasii* were 0.5, 0.015 and 0.125 μg/mL, with percentages of biofilm formation inhibition of 68.5%, 55.0% and 68.0%, respectively, compared to untreated controls. These data, along with the remaining inhibition percentages obtained at other concentrations, are available in Table 5.

**TABLE 5:** INH concentrations and their inhibitory effectiveness (%) on mycobacterial biofilm formation.

*M. tuberculosis* (ATCC 27294)		*M. tuberculosis* Clinical Isolate		*M. tuberculosis* RR Clinical Isolate		*M. kansasii* (ATCC 12478)		*M. kansasii* Clinical Isolate	
(μg/mL)	(%)	(μg/mL)	(%)	(μg/mL)	(%)	(μg/mL)	(%)	(μg/mL)	(%)
0.015	46.14	0.015	20.53	0.015	33.83	*0.015	55.03	0.015	40.61
0.03	46.84	0.03	31.29	0.03	48.91	0.03	64.85	0.03	47.94
*0.06	59.72	0.06	39.47	*0.06	63.61	0.06	76.17	0.06	48.40
0.125	70.91	0.125	47.51	0.125	78.87	0.125	81.19	*0.125	68.03
0.25	74.51	0.25	48.87	0.25	83.29	0.25	82.41	0.25	70.50
0.5	81.16	*0.5	68.53	0.5	85.99	0.5	83.80	0.5	73.11
1.0	95.30	1.0	86.33	1.0	88.75	1.0	84.63	1.0	86.99

**FIGURE 2:**
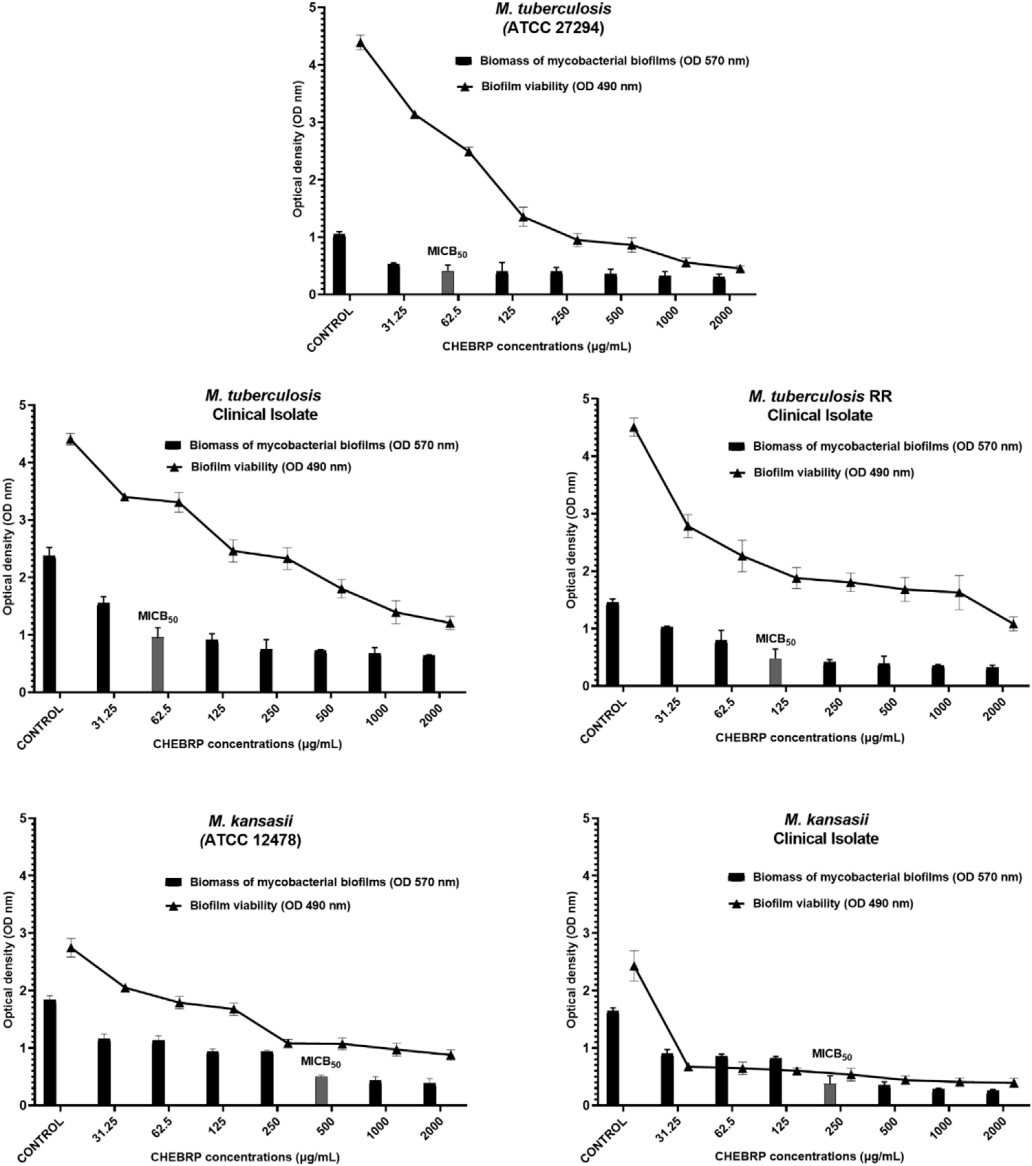
CHEBRB Biofilm Inhibition. Relation between biomass reduction (Crystal Violet) and bacterial viability (MTT) in biofilms of *Mycobacterium tuberculosis* and M. kansasii ATCC and Clinical Isolates. The mycobacteria were treated with serial dilutions of CHEBRP (ranging from 31.25 to 2,000 μg/mL). The control group corresponds to microorganisms incubated only with culture medium. Results were expressed as optical density (OD). The bars corresponding to the MICB_50_ values are highlighted. Data are presented as means ± standard deviation from experiments performed in triplicate. RR - rifampicin-resistant.

**FIGURE 3:**
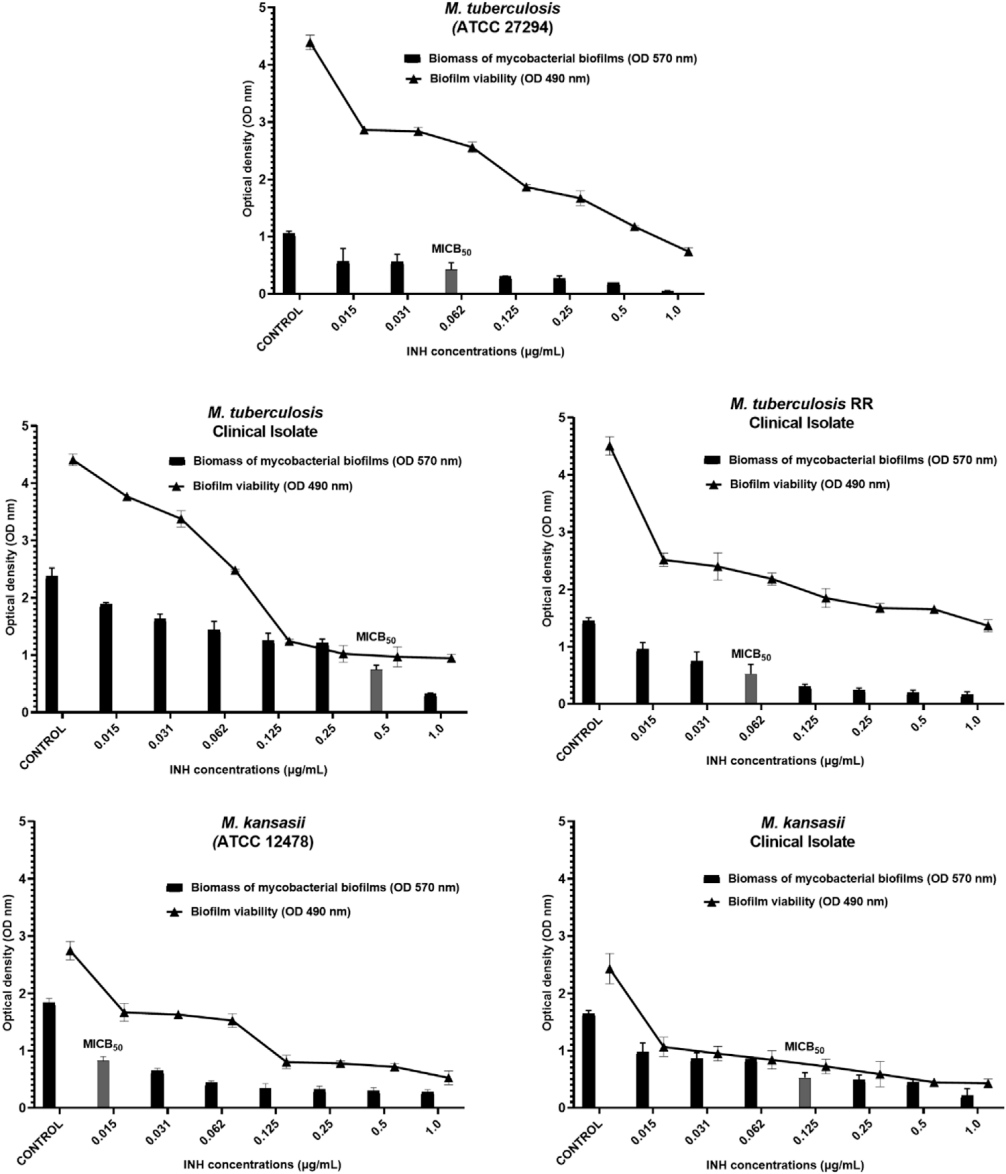
INH Biofilm Inhibition. Relation between biomass reduction (Crystal Violet) and bacterial viability (MTT) in biofilms of *Mycobacterium tuberculosis* and M. kansasii ATCC and Clinical Isolates. The mycobacteria were treated with serial dilutions of INH ranging from 0.015 to 1.0 μg/mL). The control group corresponds to microorganisms incubated only with culture medium. Results were expressed as optical density (OD). The bars corresponding to the MICB50 values are highlighted. Data are presented as means ± standard deviation from experiments performed in triplicate. RR–rifampicin-resistant.

Concomitantly with the reduction in biomass, the MTT viability assay demonstrated that both CHEBRP and INH exhibited significant and dose-dependent inhibitions in the metabolism of mycobacteria within the biofilms. These correlations can be observed in Figures 2, 3.

### Cytotoxicity test

Before evaluating the intramacrophage inhibition capacity of CHEBRP, a cytotoxicity assay was conducted using murine macrophages (Raw 264.7 lineage). The cells were exposed to CHEBRP at different concentrations (4–512 μg/mL), as well as the standard antibiotic, INH, at various concentrations (0.19–25 μg/mL).

**FIGURE 4:**
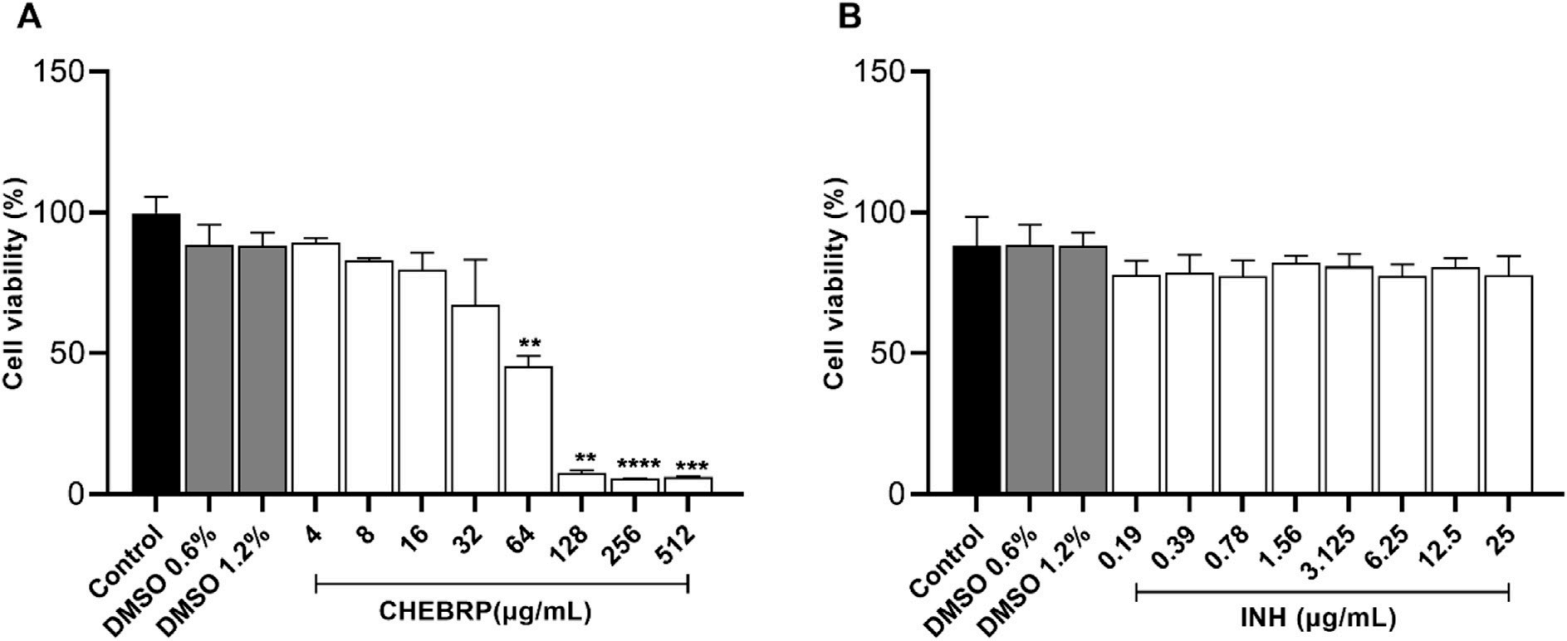
Cell viability by MTT. Raw 264.7 cells were treated for 72 h in twofold serial dilutions ranging from 4.0 to 512 μg/mL for CHEBRP **(A)** and from 0.19 to 25 μg/mL for INH **(B)**. Raw 264.7 cells were treated with culture medium only (control group) and DMSO at 0.6% and 1.2%, which are the percentages of solvent used in highest CHEBRP concentrations (256 and 512 μg/mL, respectively). Cell viability was expressed in percentage (cell viability %), with the absorbance of cells incubated only with culture medium considered as 100% viability. Data are expressed as means ± standard deviation of experiments performed in eight replicates. Significant differences detected by the Kruskal–Wallis test and Dunn’s multiple comparison test are labeled (statistically significant when P < 0.05).

As evidenced in Figure 4a, CHEBRP demonstrated cytotoxicity to macrophages at the four highest concentrations tested (64–512 μg/mL), while no significant reductions in cell viability were observed at other concentrations compared to the control. This pattern was also observed at the two concentrations of DMSO used (0.6% and 1.2%) and at all concentrations of the antibiotic INH, as illustrated in Figure 4b.

### Intracellular anti-mycobacterial activity

**FIGURE 5:**
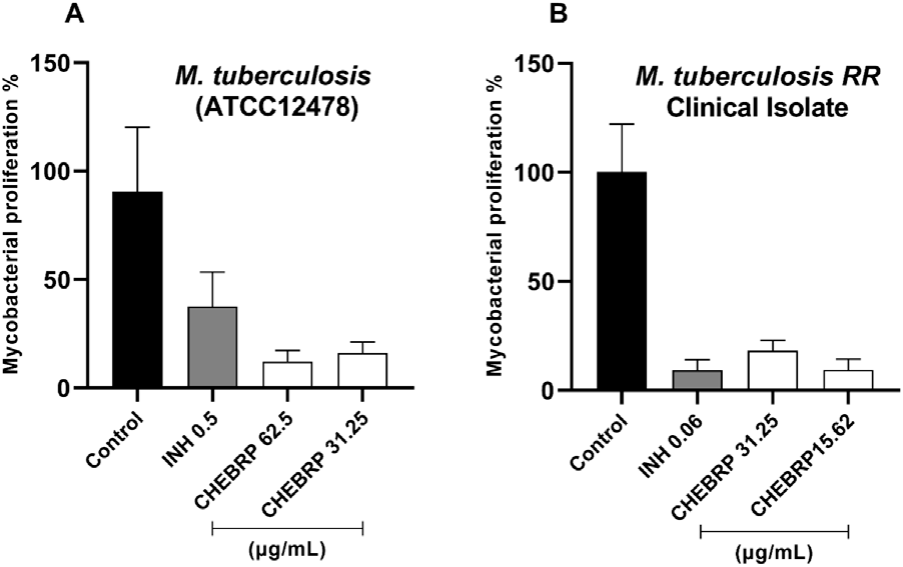
Intracellular Activity of CHEBRP in Raw 264.7. Cells Infected with *Mycobacterium tuberculosis* ATCC **(A)** and TB RR **(B)**. Raw 264.7 cells were infected (MOI 1:10), treated for 72 h with CHEBRP and INH at concentrations corresponding to the MIC and ½ MIC obtained from each strain. After 3 days, macrophages were lysed and mycobacterial viability was determined using the BacTiter-GloTM luminescence assay and expressed as a percentage (mycobacterial proliferation %), with luminescence from untreated infected cells considered as 100% viability (Control). Data are expressed as means ± standard deviation of experiments performed in six replicates. Significant differences detected by the Kruskal–Wallis test and Dunn’s multiple comparison test are labeled (statistically significant when P < 0.05).

The results of the assessment of intramacrophage activity of CHEBRP through luminescence testing demonstrated a remarkable capacity for intracellular elimination of this substance in both strains of *M. tuberculosis* evaluated and at both concentrations tested for each. The efficacy of the red propolis extract against the ATCC strain was significantly superior to that of INH, especially at the concentration of 62.5 μg/mL, equivalent to its MIC (Figure 5a). Meanwhile, the inhibitory activity of CHEBRP against *M. tuberculosis* TB RR was comparable to that of the control drug, with both showing significant inhibition (Figure 5b).

## Discussion

MIC assays are valuable for evaluating the antibacterial activity of natural products, although standardization challenges persist (Wijesundara and Rupasinghe, 2019). In this study, MIC of CHEBRP and control antibiotics were determined using the microdilution method in broth, as per CLSI (2023) M24S guidelines, and visually read after resazurin staining, following the Resazurin Microtiter Assay Plate (REMA) protocol (Palomino et al., 2007).

Interpretations of the antibacterial potential of natural products vary widely. For instance, one group interpreted MIC values according to the following criteria: results below 100 μg/mL were considered to have good antimicrobial activity; between 100 and 500 μg/mL, moderate activity; between 500 and 1,000 μg/mL, weak activity; and MIC values greater than 1,000 μg/mL were considered inactive (Holetz et al., 2002). On the other hand, some researchers accept MIC ≤200 μg/mL as indicative of good activity, especially concerning *M. tuberculosis* (Tosun et al., 2004; Nguta et al., 2016), while for another group, alcoholic plant extracts with MIC values ≤512 μg/mL show promise for the development of antituberculosis compounds (Tekwu et al., 2012).

Considering these references and comparing them with the results of this study, it is clear that CHEBRP demonstrated an overall satisfactory performance, with MIC values ranging from 31.25 μg/mL to 250 μg/mL. This highlights its promising potential for the development of antimycobacterial compounds, particularly notable in *M. tuberculosis* strains.

The literature highlights the broad antibacterial spectrum of Brazilian red propolis (BRP) against various species (Freires et al., 2016), but specific studies on its activity against the genus *Mycobacterium* were not found in the consulted databases. Our study fills this gap by providing preliminary data to understand the potential of BRP as a natural product deserving exploration for the development of new drugs capable of combating infections caused by mycobacteria.

**FIGURE 6:**
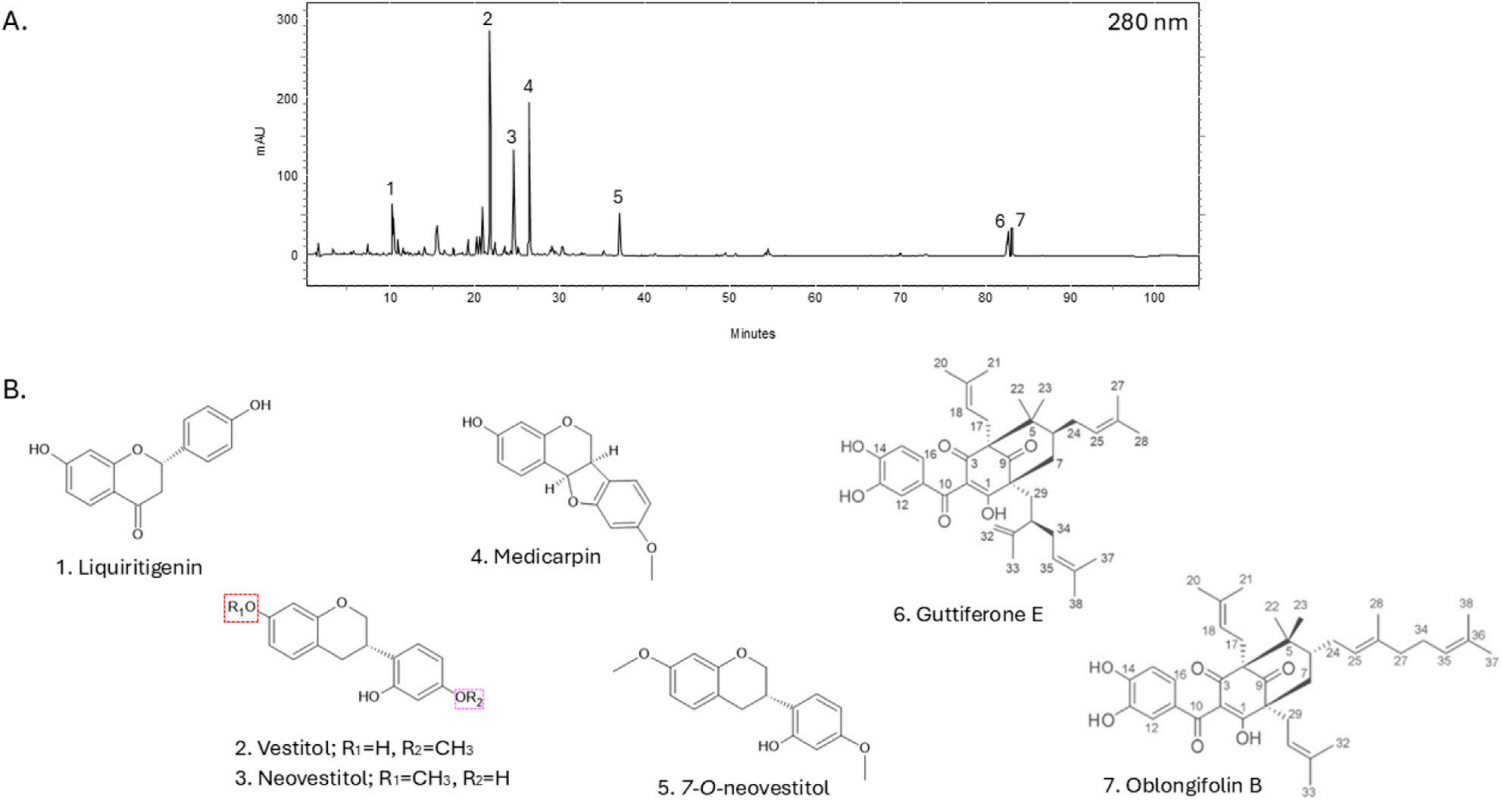
Brazilian red propolis chromatographic profile using HPLC-DAD at 280 nm absorption. Peaks are numbered **(A)** with respective chemical structures and names are shown below **(B)**.

Based on the HPLC-DAD analysis conducted on the BRP sample from “Canavieiras” (Figure 6), we believe that the compounds identified in higher concentrations, such as vestitol, neovestitol, medicarpin and polyprenylated benzophenones (guttiferone E and oblongifolin B), are primarily responsible for the antimycobacterial activity observed in our study (Bueno-Silva et al., 2013; Macedo et al., 2024; Gupta et al., 2022; Aldana-Mejía et al., 2021; De Souza Silva et al., 2021).

Previous studies have demonstrated the antimicrobial potential of guttiferone E and oblongifolin B against various bacterial and fungal pathogens, as well as their antibiofilm properties (Silva et al., 2022; 2024). These compounds have also shown efficacy in reducing biofilm formation and inhibiting intracellular proliferation of parasites like *Toxoplasma gondii*, highlighting their broad-spectrum biological activity. However, further studies using these isolated compounds are necessary to confirm this hypothesis and elucidate the specific mechanisms of action against mycobacteria.

These compounds can act in several ways, including damaging the bacterial membrane or cell wall, inhibiting cell division, reducing energy supply, altering membrane potential and modulating the immune system (Almuhayawi, 2020). Due to the growing interest in their medicinal properties, studies have documented significant interactions between propolis extracts and drugs, which can result in substantial benefits or potential clinical risks, depending on whether the effects manifest synergistically or antagonistically (Hossain S. et al., 2022). One notable case was the combination of guttiferone E with carboplatin, which demonstrated promising antitumor potential in resistant lung cancer (Nathani et al., 2024).

The synergistic effect of CHEBRP was evaluated in combination with the antibiotics INH or RIF, reference drugs indicated for all forms of tuberculosis, including new, advanced and chronic cases. The results of the associations between the red propolis extract and the drugs were indifferent. The obtained FICI values revealed that the mechanism of action of one compound did not interfere with enhancing or diminishing the effect of the other.

Although no increase in the effectiveness of the evaluated antituberculosis agents was identified when combined with CHEBRP, the results obtained in this study are relevant because the simultaneous use of propolis with other drugs may have benefits besides the synergistic action. *In vivo* studies have demonstrated that the coadministration of propolis and cefixime resulted in significant improvements in the overall condition of mice infected with *Salmonella enteric* serovar *Typhimurium*. This treatment modulated the immune system, leading to a reduction in bacterial load, an increased survival rate, normalization of hematological parameters and reduced toxicity in the kidneys, spleen and liver. The protective effect on organs was attributed to propolis and its remarkable antioxidant and free radical-neutralizing properties, corroborating that the effects of propolis reach out further than the synergistic action with antibiotics (Przybyłek and Karpiński, 2019; Kalia et al., 2016).

However, it is important to emphasize that the effects observed were only *in vitro* models, highlighting the need to confirm these results through animal studies or clinical trials in humans to determine the real effectiveness of the combination of CHEBRP with antibiotics.

In addition to investigating the antimycobacterial potential of CHEBRP alone and in combination with antibiotics against planktonic cells, this study also evaluated its ability to inhibit, *in vitro*, biofilm formation. This assessment is relevant because cells within biofilms represent an adaptation to stress, exhibiting specific genetic and protein profiles that confer upon microorganisms the ability to survive in adverse conditions, including the presence of antibiotics. Studies have demonstrated that biofilms present an adaptive resistance to most antibiotics, being up to a thousand times more effective than their planktonic forms (Davies, 2003; Hancock et al., 2021).

Moreover, these biofilms can circumvent host defense mechanisms, contributing to their ability to persist and cause chronic infections such as tuberculosis and those caused by NTM. Thus, biofilms formed by mycobacteria are the subject of intense research in microbiology due to their unique and significant characteristics, both in clinical and environmental contexts (Galié et al., 2018; Niño-Padilla et al., 2021).

Antibiofilm substances can have preventive effects, avoiding biofilm formation, or therapeutic effects, acting on established biofilms. CHEBRP demonstrated this efficacy by reducing the biomass of mycobacterial biofilms from the strains used in this study. Additionally, the MTT assay revealed that both CHEBRP and the antibiotic INH were effective in reducing the viability of mycobacteria in biofilms compared to the untreated group. Further studies indicate that the early treatment of biofilms is more effective due to the cells’ lesser adaptation to the environment. Innovative strategies are being investigated to improve the treatment of mycobacterial infections, considering the differences in biofilm development among species (Muñoz-Egea et al., 2023). In this regard, our results contribute significantly to this field.

Our study also aimed to explore the anti-tuberculosis potential of CHEBRP against the intracellular bacilli of *M. tuberculosis*, considering their ability to replicate and persist within macrophages, which protects them from certain antibiotics. Before proceeding, it was crucial to assess whether the lowest concentrations of the extract capable of inhibiting the growth of each strain were not toxic to the Raw 264.7 cells used, a widely recognized *in vitro* model for investigating the biological functions of macrophages.

Researchers identified toxicity only at concentrations above 80 μg/mL in the same cell line (Bueno-Silva et al., 2017). This finding supports the cytotoxicity analysis in this study, where only concentrations above 64 μg/mL showed a significant reduction in the viability of Raw 264.7 cells, indicating low toxicity of CHEBRP for this cell line. Regarding the cytotoxicity test, it is important to mention that control wells containing DMSO were included to rule out the possibility of cytotoxicity related to this substance, which is used as a solvent for the extract. It is noteworthy that at both DMSO concentrations of 1.2% and 0.6%, present in the highest concentrations of CHEBRP at 512 and 256 μg/mL, respectively, the observed cytotoxicity was exclusively attributed to propolis and not to the solvent used. Finally, the assessment of CHEBRP cytotoxicity through the MTT colorimetric method was of great importance, as it contributes to the selection of appropriate concentrations in future experimental studies involving Raw 264.7 cells.

Our bioluminescence assay results, which utilizes firefly luciferase to emit light in the presence of the luciferin/ATP combination and serves as a sensitive indicator of bacterial cell viability, revealed that CHEBRP demonstrated significant efficacy in eradicating intramacrophage bacilli in both strains of *M. tuberculosis* tested. These results were comparable or even superior to those observed with the antibiotic INH, known for its ability to penetrate membranes and act within phagocytic cells.

Over the years, studies have explored the effects of propolis on the immune system through various experimental approaches, highlighting its ability to modulate the function of several cells involved in innate and adaptive immunity, such as macrophages, monocytes, neutrophils, natural killer cells (NK), dendritic cells and lymphocytes, enhancing their activity and mechanisms for combating infectious agents (Sforcin, 2016). It has been demonstrated that propolis stimulated the generation of reactive oxygen species (ROS), the expression of toll-like receptors (TLR-2 and TLR-4) and the production of pro-inflammatory cytokines by murine macrophages, and also increased the fungicidal and bactericidal activity of these cells, suggesting that the product may activate mechanisms to eliminate microorganisms (Magnavacca et al., 2022). Some of the main compounds of BRP, like vestitol and neovestitol, have been shown to modulate macrophage activity by inhibiting NF-κB and ERK1/2 pathways, reducing pro-inflammatory cytokines such as interleukin-1(IL-1)-β and tumor necrosis factor (TNF)-α, shifting macrophages toward a regulatory M2-like profile (Bueno-Silva et al., 2015; Bueno-Silva et al., 2017; Bueno-Silva et al., 2020). These functions support enhanced intracellular bacterial clearance and host defense. BRP may also enhance intracellular defense mechanisms, such as autophagy, which is essential for the degradation of internalized bacteria within macrophages (Lesmana et al., 2024). These findings suggest that BRP exerts a dual mechanism of action: direct antimicrobial effects and immunomodulatory activity through the activation of host immune responses. However, further studies are needed to identify the specific compounds responsible for these effects and their mechanisms of action.

Moreover, it is noteworthy that an investigation into the effects of propolis produced in Brazil, Cuba, and Mexico showed that samples with varied chemical compositions exhibited distinct activities, potentially showing either pro or anti-inflammatory action, suggesting a relationship with differences in their botanical sources (Conti et al., 2015).

Therefore, based on this information and the results observed in this study, we can conclude that the activity against *M. tuberculosis* harbored within Raw 264.7 cells can be attributed to both the internalization and direct action of CHEBRP and the activation of macrophages by the components of the extract.

Despite the promising findings, certain limitations should be considered regarding the broader application of Brazilian red propolis. One important limitation of this study is the reliance on BRP sourced exclusively from Canavieiras, Bahia. Propolis is a chemically complex and highly variable natural product, with its composition profoundly influenced by geographical origin, botanical sources, seasonal factors, and even bee foraging behavior (Kurek-Górecka et al., 2022). Although the study acknowledges the primary plant contributors such as *Dalbergia ecastaphyllum* and *Symphonia globulifera*, it does not fully address how such variability might affect the reproducibility and therapeutic consistency of BRP. Variations in the levels of key bioactive compounds, particularly vestitol, neovestitol, and polyprenylated benzophenones, could significantly alter antimycobacterial potency, cytotoxicity, and potential synergism with antibiotics. This underscores the need for future investigations using BRP collected from multiple regions and different seasons, coupled with detailed chemical profiling and bioactivity correlation. Such efforts are essential to support standardization, ensure reproducibility, and facilitate the clinical translation of propolis-based therapies.

## Conclusion

The current study demonstrates that the crude hydroalcoholic extract of Brazilian red propolis (CHEBRP) exhibits notable *in vitro* antimycobacterial activity against both drug-susceptible and rifampicin-resistant *Mycobacterium tuberculosis* strains, as well as clinically relevant non-tuberculous mycobacteria. The extract was also effective in inhibiting biofilm formation and reducing mycobacterial viability within biofilms, alongside significant intracellular bactericidal activity in infected macrophages. Importantly, these effects were achieved with minimal cytotoxicity at therapeutically relevant concentrations.

Although the combination of CHEBRP with isoniazid or rifampicin showed no synergistic or antagonistic interaction, the retained efficacy in combination suggests its compatibility as a potential adjunctive agent. The chemical analysis confirmed the presence of several bioactive constituents, including flavonoids and benzophenones, which may contribute to the observed biological effects.

Given the promising *in vitro* results, future research should include broader screening of CHEBRP against rapidly growing mycobacteria, particularly *Mycobacterium abscessus*, a clinically challenging and emerging pathogen, to further evaluate its potential as a broad-spectrum antimycobacterial agent; as well as deeper exploration of the mechanism of action of CHEBRP, and *in vivo* studies to validate therapeutic potential, safety, and pharmacokinetics. Additionally, considering the known variability in propolis composition, it will be essential to evaluate samples from different regions and seasons, correlate their chemical profiles with bioactivity, and work toward standardized formulations to support reproducibility and clinical translation.

## Data Availability

The original contributions presented in the study are included in the article/supplementary material, further inquiries can be directed to the corresponding authors.
